# Modulation of neutrophil-to-lymphocyte ratio and gut microbiome balance in astronauts: potential benefits of novel beta-glucans during space missions

**DOI:** 10.3389/fimmu.2025.1538147

**Published:** 2025-03-03

**Authors:** Nobunao Ikewaki, Koji Ichiyama, Rajappa Senthilkumar, Senthilkumar Preethy, Samuel J. K. Abraham

**Affiliations:** ^1^ Department of Medical Life Science, Kyushu University of Medical Sciences, Nobeoka, Japan; ^2^ Institute of Immunology, Junsei Educational Institute, Nobeoka, Japan; ^3^ Antony Xavier Interdisciplinary Scholastics (AXIS), GN Corporation Co., Ltd., Kofu, Japan; ^4^ Fujio-Eiji Academic Terrain (FEAT), Nichi-In Centre for Regenerative Medicine (NCRM), Chennai, India; ^5^ Centre for Advancing Clinical Research (CACR), University of Yamanashi—School of Medicine, Chuo, Japan; ^6^ Mary Yoshio Translational Hexagon (MYTH), Nichi-In Centre for Regenerative Medicine (NCRM), Chennai, India; ^7^ Research & Development (R & D) Department, Sophy Inc., Niyodogawa, Japan; ^8^ Levy-Jurgen Transdisciplinary Exploratory (LJTE), Global Niche Corp., Wilmington, DE, United States; ^9^ Haraguchi-Parikumar Advanced Remedies (HARP) & Cherian-Yoshii Translational Exemplary (CYTE), SoulSynergy Ltd., Phoenix, Mauritius

**Keywords:** NLR (neutrophil-to-lymphocyte ratio), beta-glucans, astronauts, space mission, astronauts and nutrition

## Introduction

Among the many clinical experiments, human beings subjected to spaceflight travel stand apart because of their complexity owing to their microgravity environment. Before considering any pathological implications due to changes in the gravitational force, the fundamental physiological processes aided by Earth’s gravity are disrupted and need to be evaluated. Therefore, maintaining physiological stability despite altered gravity is the utmost priority, followed by addressing pathological implications and their management.

Spaceflight, characterized by microgravity, circadian misalignment, isolation, confinement, stress, a semi-closed food system, and increased exposure to space radiation, has been shown to have detrimental effects on the human immune system (1, 2). The immune system dysregulation reported in astronauts includes altered leukocyte distribution, changes in plasma cytokines, reduced T-cell function, and reactivation of latent herpesviruses. Persistent low-grade systemic inflammation characterized by increased TNF-α and IL-1RA levels, which can lead to various diseases, has been documented (3). The clinical implications of such immune dysfunction include rashes, hypersensitivity, atopic dermatitis, and accelerated physiological aging, as evidenced by muscle wasting and loss of bone density (1, 2).

Ongoing research has focused on identifying safe and easily evaluable in-flight biomarkers for monitoring the immune system of astronauts. The neutrophil-to-lymphocyte ratio (NLR) has been identified as a potential biomarker candidate to evaluate immune status (1, 3) because (i) leukocyte counts have been reported to be altered during spaceflight, and (ii) on Earth, elevated NLR is an extremely useful biomarker of chronic persistent subclinical inflammation, which can be a major pre-existing factor for disease development. Furthermore, an elevated NLR has been shown to predict poor prognosis in cancers and chronic conditions, such as coronary heart disease, stroke, diabetes, obesity, psychiatric diagnosis, anemia, and stress. A gradual increase in NLR, apart from having a positive correlation with age, also serves as a biomarker for predicting the overall mortality of a specific population (4).

## Significance of NLR

Recent reports (1, 3) have documented a gradual increase in NLR in astronauts, which has been suggested to be an indicator of hastened inflammation. Moreover, compared to other biomarkers of inflammation and immune status, NLR is easy to measure and has been proven to be altered under simulated spaceflight conditions on Earth, as well as in spaceflight experiments, suggesting that it is a critical biomarker for monitoring the immune system and health of astronauts (3).

NLR can be considered a critical biomarker because it acts as a bridge between innate and adaptive immune systems (5). NLR in the peripheral blood serves as a biomarker linking two key components of the immune system: innate immunity mediated by neutrophils and adaptive immunity mediated by lymphocytes. Neutrophils constitute the frontline defense of the host immune system against pathogens via mechanisms such as chemotaxis, phagocytosis, reactive oxygen species (ROS) generation, granular protein release, and cytokine production. Beyond these functions, neutrophils considerably influence adaptive immunity and are pivotal effector cells in systemic inflammatory response syndromes. As regulators of innate immunity, neutrophils recruit, activate, and modulate other immune cells by secreting diverse pro-inflammatory and immunomodulatory cytokines and chemokines, thereby enhancing the activity and recruitment of immune cells such as dendritic, B, natural killer (NK), CD4+, CD8+, γδ T, and mesenchymal stem cells (5).

However, although NLR has been identified as a critical biomarker for monitoring the immune system and prognosis of diseases both in routine clinical settings and in astronauts, safe, easily administrable dietary or nutritional interventions are still needed to beneficially modulate NLR for maintaining health in astronauts. Even in a head-down tilt bed rest experiment, considered as the best and most integrated Earth-based analog of microgravity in spaceflight, NLR was identified as a critical biomarker for astronauts and was found to increase in the study participants. However, dietary supplementation, which was part of the experiment, did not produce any changes in NLR values (3).

## Interventions to modulate NLR

Some interventions have decreased NLR in routine clinical settings. One such example is Vitamin D, wherein high-dose vitamin D supplementation reduced NLR distribution in a clinical study in adolescent girls (6). Vitamin D supplementation has been suggested as a means to beneficially modulate NLR in astronauts, and despite daily vitamin D supplementation, crew members at the Russian space station Mir had serum 25(OH)-D3 concentrations that were 32%–36% lower during and after long-duration (3- to 4-mo) missions than before the missions (7). In another study, an oral food supplement containing *Echinacea angustifolia*, rosehip, propolis, royal jelly, and zinc was shown to decrease NLR inpatients with COVID (8). Other nutritional supplements reported to influence the NLR include omega-3 fatty acids (9) and symbiotic supplements (10). However, the search continues for a dietary intervention that is safe, easy to administer, and can work on immunity, as well as other aspects contributing to optimal health, including the gut microbiome.

## Gut microbiome and NLR

Regarding the relevance of the gut microbiome to NLR and health, it is well established that 70% of immune cells in the body are found in the gastrointestinal (GI) tract, where their development and maturation are influenced by their interactions with the gut microbiota. When gut dysbiosis occurs, a clinically “maladaptive” immune response can arise (11). Changes in NLR have already been found to be directly correlated with gut dysbiosis, and gut microbiome abundance has been reported to differ considerably between patients with normal and increased NLR (12). Thus, NLR can be considered a critical indicator, along with the correlation of the gut microbiome, for monitoring health status and disease prognosis (12–14). During spaceflight and in the gut microbiome, notable changes in 44 microbiome species, including relative reductions in bile acid- and butyrate-metabolizing bacteria such as *Extibacter muris* and *Dysosmobacter welbionis*, have been reported (15). Increases in the genera *Clostridium*, *Romboutsia*, *Ruminiclostridium*, and *Shuttleworthia*, along with a decrease in *Hungatella* and significant enrichment of *Dorea* sp. and *Lactobacillus murinus*, have also been reported (16).

## Beta-glucans beneficially modulate NLR

Given the above background of several known and unknown factors that affect the health of astronauts and the critical nature of maintaining NLR and gut homeostasis, a major challenge is the result of our work on the safe and beneficial modulation of NLR in preclinical and clinical studies using the biological response modifier (BRM) Beta-1,3-1,6-glucans produced as an exopolysaccharide by the AFO-202 and N-163 strains of a black polyextremotolerant yeast, *Aureobasidium pullulans*.

Beta-Glucans are naturally occurring polysaccharides found in the cell walls of yeast, fungi (including mushrooms), certain bacteria, seaweed, and cereals such as oats and barley. These bioactive compounds possess multiple functional properties including hypocholesterolemic, hypoglycemic, immunomodulatory, antitumor, antioxidant, and anti-inflammatory activities. The structural and functional properties of beta-glucans vary depending on their sources. Among the various types, yeast-derived beta-1,3-1,6-glucans have demonstrated superior BRM effects compared to those derived from cereal sources, such as oats or barley. Clinical applications of beta-glucans have gained prominence worldwide, with Japan being the leader in their therapeutic utilization (17). Since 1983, beta-glucans derived from *Lentinula edodes* (lentinan) and *Coriolus versicolor* (polysaccharide-K) have been approved as pharmaceutical agents. As of 2019, more than 177 clinical trials have been registered in the United States, evaluating the potential of beta-glucans in cancer therapy, cholesterol regulation, and immune modulation (18). The immunomodulatory properties of beta-glucans stem from their ability to interact with a range of immune receptors, including Dectin-1, complement receptor 3, lactosylceramide, natural cytotoxicity receptor p30, and scavenger receptors. These receptors are expressed on key immune cells such as macrophages, neutrophils, and NK cells, enabling beta-glucans to modulate immune responses effectively. Owing to their capacity to either enhance or regulate immune functions, beta-glucans have been extensively explored as potential therapeutic adjuvants, particularly in immunotherapy. Extensive research has established that beta-glucans produced as exopolysaccharides by two novel strains of black yeast (*A. pullulans*), AFO-202 and N-163, exhibit unique immunomodulatory and metabolic-immune-enhancing benefits (17).

AFO-202 beta-glucan has been shown to reduce NLR in Sprague–Dawley rats (19). In a 30-day study involving patients with COVID-19, a 70% reduction from baseline was observed in the group administered AFO-202 beta-glucan. In the group receiving a combination of AFO-202 and N-163 beta-glucan, a 66% reduction was observed from baseline (20). Apart from NLR reduction, a decrease in other inflammatory markers, such as IL-6 and D-dimer, and an increase in the lymphocyte-to-C-reactive and leukocyte-to-C-reactive protein ratios were observed (20). In patients with pancreatic cancer undergoing surgery, perioperative administration of AFO-202 led to a 40% reduction in NLR (21). The clinical outcomes were decreased serum amyloid A, sCD44, and CA19-9 levels and increased mean survival time (21). AFO-202 beta-glucan has been shown to have anti-infective properties against *Leishmania amazonensis* and malaria through an increase in NK cell activity and cellular immunity (22) and has also shown potential as a vaccine adjuvant, enhancing the immune response to avian influenza A H5N1 and H5N2 vaccines (23). N-163 produced beta-glucan has been reported to attenuate lipotoxicity, as evidenced by a decrease in non-esterified fatty acids (24), with anti-inflammatory and anti-fibrotic effects in animal and human clinical studies of metabolic dysfunction-associated diseases such as nonalcoholic steatohepatosis (NASH) (25) and Duchenne muscular dystrophy (DMD) (26, 27).

In the gut microbiome, AFO-202 beta-glucan in children with autism spectrum disorder has been shown to decrease the abundance of harmful *Enterobacteriaceae*, including *Escherichia coli*, *Akkermansia muciniphila* CAG:154, *Blautia* spp., *Coprobacillus* sp., and *Clostridium bolteae* CAG:59, with an increase in butyrate producers, such as *Faecalibacterium prausnitzii* and *Roseburia* (28). In the NASH model (29), gut microbial diversity increased greatly in the AFO-202 + N-163 group. In the AFO-202 + N-163 group, the abundance of *Firmicutes* decreased, whereas those of *Bacteroides* and *Lactobacillus* increased. In NASH mice fed AFO-202 beta-glucan alone, there was a decrease in the abundance of *Enterobacteriaceae* and other *Firmicutes*, whereas in the N-163 group, there was a decrease in the abundance of harmful bacteria, such as *Turicibacter* and *Bilophila*. In the same study, an increase in the abundance of butyrate precursors and amino acids, such as tryptophan, has also been reported (29, 30). The administration of N-163 produced beta-glucan, resulting in an increase in butyrate-producing species, such as *Roseburia* and *F. prausnitzii*, and a decrease in harmful bacteria associated with inflammation, such as *Enterobacteria* and *Alistipes*, in patients with DMD. In patients with multiple sclerosis, there is an increase in the abundance of beneficial genera such as *Bifidobacterium*, *Collinsela*, *Prevotella*, and *Lactobacillus*, as well as species such as *Prevotella copri*, *Bifidobacterium longum*, *F. prausnitzii*, and *Siphoviridae*, whereas there is a decrease in inflammation-associated genera such as *Blautia*, *Ruminococcus*, and *Dorea* (31).

## Discussion

**Figure 1:**
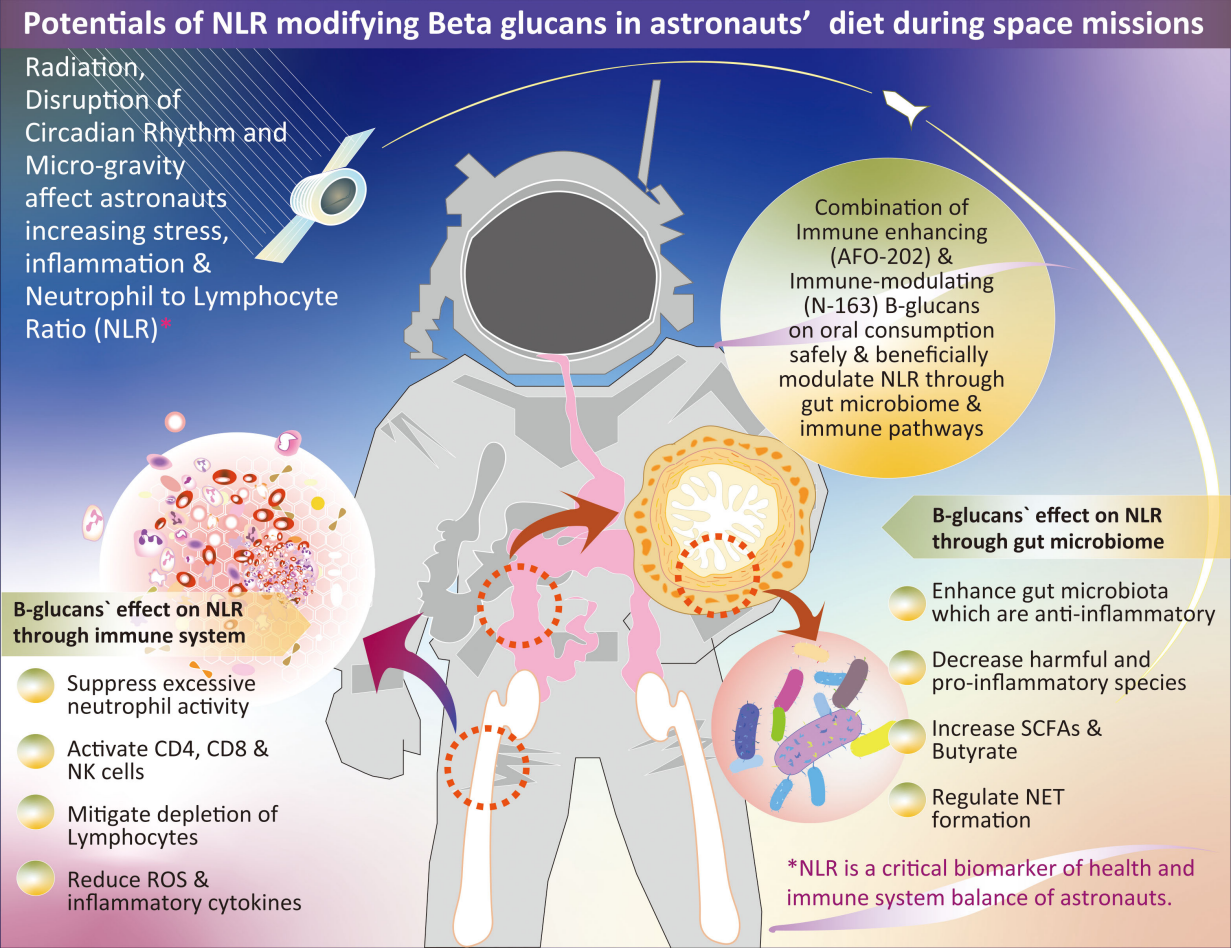
Overview of the potential role of NLR-modifying beta-glucans in astronaut diets during space missions. The figure illustrates the mechanisms through which these beta-glucans influence NLR modulation by impacting the immune system and gut microbiome; NLR, neutrophil-to-lymphocyte ratio; SCFA, short-chain fatty acids; NET, neutrophil extracellular trap.

These findings on the novel beta-1,3-1,6–glucan-based BRMs produced from the AFO-202 and N-163 strains of *A. pullulans* align with astronaut requirements. As mentioned earlier in this manuscript, studies have documented increases in the NLR and imbalances in microbial species during space missions (1, 3, 15, 16), which have been reported to be beneficial in studies using these beta-glucans (17–31). The beneficial effects of lowering the NLR are primarily mediated through the suppression of excessive neutrophil activity and restoration of balanced lymphocyte function (32). The mechanisms underlying the improvement in disease outcomes include (1) Neutrophilia reduction because elevated neutrophil counts, which are common in chronic inflammatory conditions, are linked to excessive ROS production, prolonged inflammation, and tissue damage. Lowering NLR reduces neutrophil-driven inflammation and oxidative stress. (2) NETosis regulation, where excessive neutrophil extracellular trap (NET) formation, which is implicated in microvascular complications in diabetes mellitus, atherosclerosis in coronary artery disease, and airway damage in chronic obstructive pulmonary disease (COPD), is attenuated with decreased NLR, reducing endothelial and tissue injury (32). (3) Decreased pro-inflammatory cytokine secretion: Decreased neutrophil burden limits the release of TNF-α, IL-6, and other pro-inflammatory mediators, thereby curbing chronic systemic inflammation. (4) Enhanced lymphocyte recruitment to inflamed sites: A balanced NLR ensures optimal lymphocyte trafficking, aiding in effective immune surveillance and resolution of inflammation (32). (5) Treg and Th17 balance restoration: Decreased NLR is associated with increased regulatory T cell (Treg) activity, which suppresses excessive inflammation and reduces Th17-driven autoimmunity, contributing to chronic inflammatory pathology. (6) Improved adaptive immune response: Lymphocytopenia, which is linked to immune dysfunction, is mitigated by lowering NLR, enhancing antigen-specific immune responses, and reducing infection susceptibility. Thus, lowering NLR contributes to immune homeostasis by regulating neutrophil overactivation and enhancing lymphocyte function (32). This balance is critical for mitigating chronic inflammation, improving disease prognosis, and reducing complications of various inflammatory conditions. Thus, *A. pullulans* AFO-202 and N-163 produce beta-glucans, with a long safety track record as a food supplement, which are water soluble and produced with any ingredient in the commonly notified list of allergens and are promising candidates for consideration in nutrition studies for astronauts and in space flight experiments because of their beneficial NLR-modulating effects in a safe manner (17, 19–31). **Figure 1** summarizes the potential of NLR-modifying beta-glucans in astronaut diets during space missions. It also illustrates the possible mechanisms through which NLR modulation occurs, highlighting the effects of these beta-glucans on the immune system and gut microbiome.

These novel NLR-modifying beta-glucans’ ability to exert beneficial immune modulation, as observed in studies involving healthy human volunteers (33), further supports their potential, after necessary validation for inclusion in astronaut diets as a routine intervention. Despite experiencing physiological stressors, such as microgravity, circadian misalignment, and space radiation exposure, astronauts differ considerably from patients with chronic diseases and healthy terrestrial subjects. Unlike individuals with preexisting inflammatory or metabolic conditions, astronauts are highly trained and undergo rigorous physical and psychological preparation, which may influence their immune adaptability and inflammatory responses. Therefore, although the observed effects of *A. pullulans* AFO-202 and N-163 beta-glucans on immune regulation, particularly in modulating NLR and systemic inflammation, align with the physiological challenges encountered during spaceflight, further research is warranted to validate these findings, specifically in the astronaut population. Controlled studies of spaceflight-relevant models and actual space missions are required to establish the extent of their benefits in the unique physiological environment of space.
